# Use of implementation mapping in the planning of a hybrid type 1 pragmatic clinical trial: the BeatPain Utah study

**DOI:** 10.1186/s43058-023-00542-z

**Published:** 2024-01-05

**Authors:** Julie M. Fritz, Bryan Gibson, David W. Wetter, Guilherme Del Fiol, Victor Solis, Isaac Ford, Kelly Lundberg, Anne Thackeray

**Affiliations:** 1 Department of Physical Therapy & Athletic Training, University of Utah, 383 Colorow Dr., Room 391, Salt Lake City, UT 84108 USA; 2 Department of Biomedical Informatics, University of Utah, Salt Lake City, UT USA; 3 Department of Population Health Sciences, Center for Health Outcomes and Population Equity, Huntsman Cancer Institute, University of Utah, Salt Lake City, UT USA; 4 Department of Psychiatry, University of Utah, Salt Lake City, UT USA

## Abstract

**Background:**

Considerable disparities in chronic pain management have been identified. Persons in rural, lower income, and minoritized communities are less likely to receive evidence-based, nonpharmacologic care. Telehealth delivery of nonpharmacologic, evidence-based interventions for persons with chronic pain is a promising strategy to lessen disparities, but implementation comes with many challenges. The BeatPain Utah study is a hybrid type 1 effectiveness-implementation pragmatic clinical trial investigating telehealth strategies to provide nonpharmacologic care from physical therapists to persons with chronic back pain receiving care in ommunity health centers (CHCs). CHCs provide primary care to all persons regardless of ability to pay. This paper outlines the use of implementation mapping to develop a multifaceted implementation plan for the BeatPain study.

**Methods:**

During a planning year for the BeatPain trial, we developed a comprehensive logic model including the five-step implementation mapping process informed by additional frameworks and theories. The five iterative implementation mapping steps were addressed in the planning year: (1) conduct needs assessments for involved groups; (2) identify implementation outcomes, performance objectives, and determinants; (3) select implementation strategies; (4) produce implementation protocols and materials; and (5) evaluate implementation outcomes.

**Results:**

CHC leadership/providers, patients, and physical therapists were identified as involved groups. Barriers and assets were identified across groups which informed identification of performance objectives necessary to implement two key processes: (1) electronic referral of patients with back pain in CHC clinics to the BeatPain team and (2) connecting patients with physical therapists providing telehealth. Determinants of the performance objectives for each group informed our choice of implementation strategies which focused on training, education, clinician support, and tailoring physical therapy interventions for telehealth delivery and cultural competency. We selected implementation outcomes for the BeatPain trial to evaluate the success of our implementation strategies.

**Conclusions:**

Implementation mapping provided a comprehensive and systematic approach to develop an implementation plan during the planning phase for our ongoing hybrid effectiveness-implementation trial. We will be able to evaluate the implementation strategies used in the BeatPain Utah study to inform future efforts to implement telehealth delivery of evidence-based pain care in CHCs and other settings.

**Trial registration:**

ClinicalTrials.gov Identifier: NCT04923334. Registered June 11, 2021.

Contributions to the literature

- Increasing access to evidence-based, nonpharmacologic pain care through telehealth is a promising strategy to reduce disparities, but careful attention to the unique implementation challenges in historically marginalized communities and low-resource healthcare settings is critical.
- We used implementation mapping, informed by additional frameworks and models, to systematically develop a multifaceted implementation plan for a hybrid type 1 effectiveness-implementation pragmatic clinical trial being conducted in community health centers.
- Implementation mapping provides a comprehensive strategy to gather stakeholder input, develop tailored implementation strategies, and identify implementation outcomes. Implementation mapping is particularly well-suited to planning a hybrid pragmatic clinical trial.

## Introduction

One in 5 Americans live with chronic pain [1]. Back pain is the most prevalent form of chronic pain [2]. Evidence-based interventions (EBIs) for chronic low back pain (LBP) include nonpharmacologic treatments provided by physical therapists (PTs) and others [3, 4]. Guidelines advise against opioids [5–7], yet evidence-practice gaps persist. Among persons with LBP, rates of opioid prescribing are about double rates of nonpharmacologic care [8–14].

Considerable disparities exist in chronic pain prevalence and management. Prevalence is higher among persons with less income or education and in rural communities [15, 16]. These individuals are more likely to receive opioids [17–19] and less likely to receive nonpharmacologic care [20, 21]. Many persons with these characteristics receive primary care in community health centers (CHCs) [22–24], yet geographic and other barriers limit access to nonpharmacologic care [23]. About a third of CHC patients are Latino/a, introducing additional barriers related to language and sociocultural fit [25, 26]. Collectively, these barriers contribute to pain disparities [27].

Telehealth delivery of nonpharmacologic care could lessen disparities [28], but application has been limited [29, 30]. Experiences during COVID support telehealth’s potential to increase access [31], but issues specific to implementation in underserved communities must be considered [32]. Implementation mapping (IM) is a systematic approach to iteratively develop scalable and sustainable EBI implementation strategies [33, 34]. This paper describes application of IM in the BeatPain Utah study examining telehealth EBIs for patients with chronic LBP in CHCs.

## Methods

### BeatPain Utah study

**Fig. 1:**
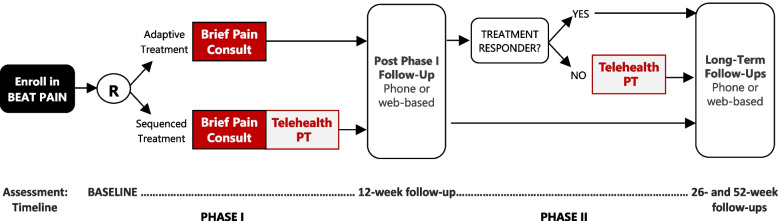
BeatPain study design

BeatPain Utah (Fig. 1) (ClinicalTrials.gov Identifier: NCT04923334) is a pragmatic clinical trial examining two PT-led telehealth EBIs, a brief consult and an extended PT program, delivered across two treatment phases. Further details are published [35]. BeatPain is a hybrid type 1 trial, primarily focusing on effectiveness and secondarily on implementation outcomes [36]. Implementation mapping occurred during a planning year before enrollment. The implementation strategies developed are being evaluated as part of the ongoing hybrid clinical trial. Planning activities, including community-member interviews, were approved by the University of Utah Institutional Review Board.

BeatPain Utah is being conducted in nine CHC organizations in Utah serving urban and rural communities. Among persons served by these clinics, approximately 49% identify as Hispanic/Latino/a, 37% are non-English speakers, 45% are uninsured, and 59% are below the federal poverty level [37]. Patients in CHC clinics are referred to BeatPain through standards-based, HIPAA-compliant electronic referral (e-referral) from a CHC EHR using phiMail® (EMR Direct, Inc., San Diego, CA, USA). Once an e-referral is received, a BeatPain team member contacts the patient. Patients opting to enroll provide oral consent. Those choosing not to participate or ineligible are offered care without study data collection.

### Implementation logic model

**Fig. 2:**
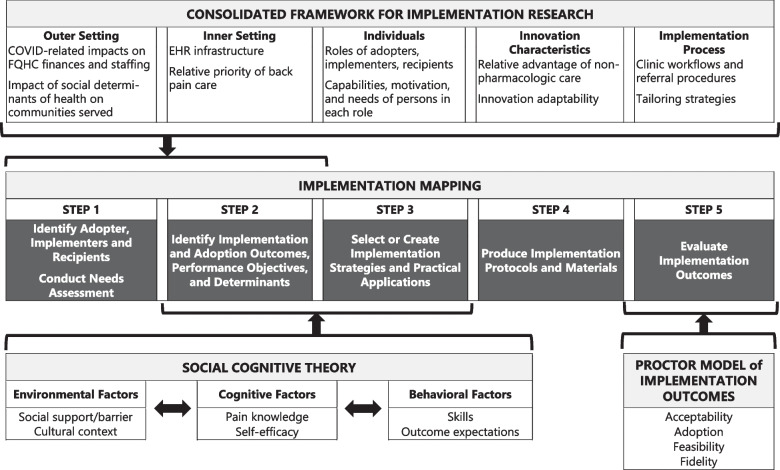
Comprehensive logic model for the implementation of e-referrals and telehealth physical therapy

Implementation mapping was informed by additional frameworks to develop a logic model (Fig. 2). The Consolidated Framework for Implementation Research (CFIR) helped identify contextual factors across five domains that could influence e-referral and telehealth implementation: the innovation being implemented, outer setting, inner setting, individuals involved, and the implementation process. Social-cognitive theory (SCT) emphasizes factors influencing behavior including the following: (1) environmental factors (e.g., social support, cultural context); (2) cognitive factors (e.g., knowledge, self-efficacy); and (3) behavioral factors (e.g., coping strategies, outcome expectancies) [38]. Proctor’s taxonomy of Outcomes for Implementation Research [39], informed IM step 5.

### Implementation mapping

Implementation mapping was based on a five-step model [33] detailed below and depicted in Fig. 2.

#### Step 1 — Needs assessment

Step 1 identified barriers and facilitators using needs assessment for groups involved in implementation including patients, CHC leadership/providers, and PTs [40, 41]. For CHC leadership/providers, needs assessments were conducted using zoom due to COVID restrictions. Assessments focused on the existing and desired clinic workflows for identifying and treating individuals with LBP, and the type of feedback desired about referred patients. The goal was collection of data to inform e-referral implementation [42, 43]. For patients, we conducted individual, zoom interviews with individuals with LBP in communities served by CHC clinics identified by working with community organization and clinic representatives. Interviews used an ethnographic approach focused on persons’ lived experience including their understanding of LBP and healthcare experiences using questions described by Blumenthal and colleagues [44]. Participant responses were noted in a REDCap. Needs assessments for PTs about telehealth implementation were conducted in group zoom meetings.

#### Step 2 — Identify implementation outcomes, performance objectives, and determinants

Step 2 used step 1 findings to identify implementation outcomes and performance objectives for each group. Implementation outcomes identified behavior goals. Performance objectives identified tasks required to achieve an implementation outcome. Next, determinants were specified, informed by step 1 findings and the SCT framework [45]. Determinants are modifiable, internal factors helping explain why persons would achieve a performance objective [46]. For example, self -efficacy is an important determinant of behavior change and relates to one’s beliefs in their capacity to perform an action and persist despite barriers [38]. Another behavior change determinant is outcomes expectancy, i.e., is the belief that performing an action will lead to a particular outcome, and is another behavior change determinant [47].

#### Step 3 — Select implementation strategies

In step 3, methods or processes that can change a determinant based on evidence or theory (e.g., increase knowledge, change attitudes) [48] were selected. Methods informed the choice of implementation strategies. Strategies [49] were then operationalized as practical applications that fit within the context described by CFIR and the needs assessments [46].

#### Step 4 — Create implementation protocols

Step 4 operationalized implementation strategies by producing protocols and materials for training and delivery.

#### Step 5 — Evaluate implementation outcomes

Step 5 developed a plan to evaluate outcomes of the implementation processes during the BeatPain study. Outcomes were based on Proctor’s taxonomy [39] and included acceptability, adoption, feasibility, and fidelity.

## Results

### Step 1 — Needs assessment

Needs assessment outcomes are described in Table 1. Assessments were conducted with CHC leaders/providers from seven organizations. Participants expressed receptivity to PT-led telehealth. Facilitators included the ability to accommodate patients in Spanish or English, without cost, and receiving feedback about referred patients. Potential barriers centered on EHR capabilities for making e-referrals, time constraints and competing demands, and integrating a new workflow, particularly in light of COVID impacts on clinic operations.

**Table 1:** Results of the needs assessments conducted for implementation mapping step 1 across groups (*CHC*, community health center; *EHR*, electronic health record)

Group	Assessment method	Facilitators	Barriers
CHC leadership and providers	Sociotechnical group interviews with CHC organizations conducted remotely	Supportive of providing nonpharmacological pain management options Recognition of the impact of the opioid epidemic within community being served Availability of service in English or Spanish to accommodate patients Ability to receive feedback on persons referred from the BeatPain team	Time constraints and competing demands during patient care Limited understanding of clinic staff about EHR capabilities and how to send e-referrals Staffing shortages, COVID restrictions, and turnover make consistent work flows challenging Lack of a systematic process for managing patients with chronic LBP
Patients	Individual zoom interviews with CHC patients with chronic back pain	All patients had smart phones and Internet access Patients trusted CHC clinics and providers Most patients did not perceive language or culture as barriers to care provided by CHCs	Concerns about treatment access due to costs Most had not heard of telehealth physical therapy and were not certain it would help Preference for passive pain care (rest, medication, etc.)
Physical therapists	Group discussions with physical therapists	Strong commitment to reducing pain management disparities and provide an accessible, nonpharmacologic pain management option Prior experience providing care in low-income and Spanish-speaking communities Availability of team members with Spanish language skills and cultural background	Limited experience or training to provide treatments using telehealth Inexperienced providing care using audio-only telehealth delivery with no video access Concerns about the ability to engage and motivate patients and provide effective exercise instruction via telehealth

We interviewed five female patients with LBP, each of whom identified as Hispanic/Latina. Three preferred communications in Spanish and two in English. Facilitators identified included positive experiences and trust in CHCs and availability of cell phones for telehealth. Barriers included lack of reliable Internet and technology for video telehealth sessions. There was a general lack of awareness that PT could be provided by telehealth, and that it may be beneficial. Some interviewees expressed preferences for passive pain coping, including medication or rest, which are not EBIs.

Facilitators for PTs included commitment to providing care to persons in historically marginalized communities. Most were bilingual, and some had experience providing PT with Spanish-speaking patients. Barriers included lack of telehealth experience, the need to adapt treatments for phone-only and video telehealth delivery, and ability to engage and motivate patients using telehealth.

### Step 2 — Identify implementation outcomes, performance objectives, and determinants

We identified implementation outcomes as participating in BeatPain and referring persons with LBP for CHC leadership/providers, engaging in telehealth for patients, and providing telehealth with fidelity to intervention core components for PTs. Performance objectives derived from these outcomes, and associated determinants are outlined in Table 2.

**Table 2:** Findings from implementation mapping steps 2 and 3 across groups (CHC, Community Health Center; EHR, electronic health record; ⥥implementation strategies based on the taxonomy of Waltz et al. [49])

Group	Performance objectives	Determinants	Implementation strategies⥥	Practical applications
CHC leadership and providers	Agree to participate in BeatPain	Knowledge of benefits of nonpharmacologic care for chronic back pain Knowledge of the BeatPain program	Train and educate stakeholders	Provide evidence supporting nonpharmacologic care for chronic back pain and potential benefits of telehealth delivery
	Ask and advise patients about BeatPain program during primary care visits	Self-efficacy to advise patients on non-pharmacologic care Positive expectations for outcomes of physical therapy	Provide ongoing consultation Support clinicians Feedback clinical data	Provide initial and ongoing training on BeatPain and the e-referral process Provide feedback to clinicians on status of referred patients and their outcomes
	Refer interested patients to BeatPain	Skills to generate e-referral from EHR and manage technology-based issues that arise	Change the infrastructure Provide local technical assistance	E-referral process embedded in EHR Ongoing technological support for issues that arise during the study
	Assist in sustaining clinic participation in BeatPain	Positive expectations for clinical workflows from e-referral process	Provide interactive assistance to avoid disruptions in workflows	Patient outreach using text messaging after in-clinic visits if in-clinic e-referral is not made
Patients	Provide consent for BeatPain study	Knowledge of telehealth physical therapy as a pain care option	Prepare consumers to be active participants	Education on BeatPain program and potential benefits of telehealth physical therapy
	Attend BeatPain telehealth sessions	Positive expectations for outcomes of telehealth physical therapy		
	Engage in BeatPain treatments	Self-efficacy for active pain coping strategies	Adapt and tailor to context	Use of motivation and problem-solving strategies to provide telehealth physical therapy
Physical therapists	Attend PT trainings	Knowledge of strategies to provide care via telehealth	Create a clinical team Develop educational materials Conduct ongoing training	Create training materials and procedures for ongoing training on telehealth delivery. Integrate mHealth resources
	Incorporate cultural competencies with BeatPain core components	Self-efficacy for providing BeatPain core components in a patient-centered manner		Training on cultural competencies for providing care
				Training to integrate motivation and problem-solving strategies with the core components of BeatPain treatments
	Provide patient-centered care			Regular team meetings for problem-solving and skills practice

Determinants informed our choice of implementation strategies to help each group achieve their performance objectives. For CHC leadership/providers, we identified knowledge of nonpharmacologic pain care and expectations for telehealth PT as determinants of agreeing to participate and place e-referrals. The ability to provide technical support for the implementation of e-referrals within clinic EHRs and avoid workflow disruptions was determinants of sustainment of e-referrals. For patients, we considered knowledge and outcomes expectancy for telehealth PT as determinants for engaging in BeatPain. Determinants for PTs included knowledge of how to deliver the BeatPain intervention core components using telehealth and self-efficacy to engage patients who may have different cultural backgrounds.

### Step 3 — Select implementation strategies

Implementation methods relevant across groups included increasing knowledge, changing awareness, changing attitudes and beliefs, and developing skills, capabilities and self-efficacy, and outcome expectations [48]. We matched these methods to implementation strategies (Table 2).

Implementation strategies for CHC leadership/providers included education about nonpharmacologic LBP care and the BeatPain program and training on how to explain BeatPain to patients to increase self-efficacy for referring patients. Additional implementation strategies included hands-on technical support to implement e-referrals within each clinic’s EHR (three different EHR products are used across organizations) and provide ongoing technical support. We used a secure, EHR-based process designed to be minimally disruptive to existing workflows. We chose secure messaging based on the direct standard protocol using phiMail because this approach was HIPAA compliant, standards-based, inexpensive, and bidirectional. Since the Direct Protocol is required for EHR certification, it was supported by all EHR systems used in Utah CHC clinics [50]. This strategy also provided the infrastructure for PTs to return feedback to providers on patients’ status, helping to build positive expectations about BeatPain.

Although in-clinic e-referrals are minimally disruptive, they require clinician recall during a visit. We therefore included a second implementation strategy using text messaging to capture individuals for whom a referral may have been unaddressed during the visit. We used a population health management system (Azara Healthcare, Burlington, MA, USA) that interfaces with clinic EHRs and identifies eligible patients (recent appointment for LBP) and then automatically sends a bidirectional text message introducing the BeatPain project and offering a connection to telehealth. Patients who respond positively are noted on an electronic dashboard, and clinic staff can place an e-referral.

Implementation strategies for patients focused on engagement to build positive expectancies for telehealth PT. Explanations used by BeatPain personnel to describe the program to patients and patient-facing materials such as the project’s webpage were tailored to provide information on what telehealth PT involves and its potential benefits. Additionally, a strategy of adapting and tailoring telehealth PT interventions addressed the determinant of building patient self-efficacy for active pain coping. Telehealth PT interventions were adapted to include a motivation-and-problem-solving (MAPS) approach found effective for chronic care management and substance use treatment [51]. The MAPS approach is appropriate for persons irrespective of their readiness to change and explicitly targets motivation and self-efficacy as behavior change mechanisms [52, 53].

Physical therapist implementation strategies included training and education on providing care using telehealth, especially when communication is audio-only. Training included didactic information, role-playing, ongoing weekly discussions, and peer practice. Integration of mHealth resources including app-based exercise and education platforms helped support patients’ self-management. Physical therapists were trained in MAPS using didactic and interactive strategies to build self-efficacy for delivering the BeatPain core components. MAPS includes motivational interviewing and cognitive behavioral techniques that help patients set personalized goals and manage barriers towards achieving these goals [51]. To further build self-efficacy, the MAPS expert on the BeatPain team provided coaching through role-playing a PT session and providing feedback. Physical therapists were also trained on culturally competent care to meet patients’ sociocultural and linguistic needs [54].

### Step 4 — Create implementation protocols

Step 4 operationalized implementation strategies. For CHC leadership/providers, we developed brief (10–15 min) presentations, and for in-person or remote delivery, focused on evidence supporting nonpharmacologic care and the BeatPain program. Detailed instructions for placing e-referrals in the clinic’s EHR were provided along with suggested language providers could use to describe BeatPain to patients. Ongoing updates were provided during staff meetings, including reminders on e-referral procedures, troubleshooting barriers, and anecdotal patient experiences.

We operationalized patient implementation strategies by developing a study webpage, in English and Spanish, accessible through a QR code on recruitment materials, to describe BeatPain and build positive expectations (https://health.utah.edu/physical-therapy-athletic-training/research/clinical-outcomes-researh/beatpain-utah/eng). The webpage described the partnership between BeatPain and CHC clinics, gave biographies of BeatPain personnel, and described telehealth PT treatment. We used the MedBridge phone app (MedBridge, Inc., Bellevue, WA, USA) to provide exercise and education videos.

Physical therapist training focused on the intervention core components and integration of MAPS for English- and Spanish-speaking patients [35]. Training in culturally competent care used Hays ADDRESSING framework as a structured self-exploration method of how the PT’s cultural background may interact with their patient’s background and influence care [55]. We also used Betancourt’s framework for cross-cultural communication to help PTs consider major cultural issues they may encounter and provide person-centered care characterized by empathy and respect for patients’ values and preferences [56].

### Step 5 — Evaluate implementation outcomes

Implementation outcomes for the BeatPain trial [35] were selected to evaluate the success of the IM process across groups. Because BeatPain adapted nonpharmacologic pain EBIs for underrepresented populations using novel delivery strategies, we identified important implementation outcomes as adoption, acceptability, feasibility, and fidelity as defined in Table 3.

**Table 3:** Implementation outcomes evaluated in the BeatPain study (CHC, community health center; ^a^outcome domains and definitions based on the framework of Proctor et al. [39]). Of note, the performance objectives in Table 2 were identified as the critical steps for achieving the implementation outcomes listed here

Implementation outcome domain^a^	Group	Definition^a^	Operationalization
Acceptability	Patients	Perception that a treatment is agreeable or satisfactory	Percentage of patients asked about BeatPain who agree to participate
Adoption	CHC provider	The intention or action to employ a new treatment	Percentage of potentially eligible patients who are asked about BeatPain
Feasibility	Patients	The extent to which a new treatment can be successfully carried out	Percentage of intervention sessions that are completed by patients
Fidelity	Physical therapists	The degree to which an intervention is implemented as intended	Percentage of core components of BeatPain treatments that are provided

## Discussion

Implementing telehealth pain care in CHCs creates an opportunity to increase the reach of EBIs and reduce pain management disparities. BeatPain Utah uses an e-referral process of persons with LBP from CHC clinics to a centralized telehealth PT team, requiring behavior changes and new work processes for clinical teams, patients, and PTs. The five-step IM process informed by additional models helped us to understand needs and assets for CHC leadership/providers, patients, and PTs, identify actions necessary to achieve implementation outcomes, identify determinants of those actions, and operationalize implementation strategies to address key determinants. Through this process, we developed a multifaceted implementation plan to connect patients with telehealth EBIs. The final IM step identified implementation outcomes for the hybrid effectiveness-implementation study.

We identified knowledge and positive expectations around nonpharmacologic pain care, EHR support for placing e-referrals, and minimizing workflow disruptions as important determinants of e-referrals from CHC clinics, consistent with other findings that technology challenges and workflow disruptions are barriers to e-referral implementation and sustainment [57]. These determinants were influenced by COVID impacts on staffing and the varied EHR systems used within clinics. We used brief, intermittent trainings on the e-referral process, ongoing EHR support, and a secondary text message recruitment approach as strategies to address these determinants. Lack of knowledge and uncertain expectations for telehealth PT were not surprising given limited exposure to nonpharmacologic EBIs or telehealth PT in CHCs [20, 23, 58] but could adversely impact providers’ self-efficacy for advising patients with LBP about BeatPain. Educating providers and sending feedback on referred patients addressed these concerns.

Patient needs assessments reflected uncertainty that telehealth PT is equivalent to in-person care, consistent with other reports [59, 60]. Also, studies during COVID reported persons of Hispanic ethnicity expressed less willingness to use telehealth [61, 62]. We therefore considered positive expectations about telehealth PT, a determinant of patients’ attendance, which we addressed by emphasizing the personalized nature of telehealth and ability to individualize care in patient-facing materials. Some patients expressed preferences for passive coping strategies that are associated with lower self-efficacy for engaging in active EBIs such as physical activity [63]. Developing self-efficacy for active coping had to balance recognition that preferences can reflect cultural norms and individual experiences [64–66]. We addressed this through training PTs in cross-cultural communication emphasizing the patient as teacher and strategies to negotiate differences in a patient-centered manner [56, 67].

Like most PTs, BeatPain therapists had limited telehealth experience [68]. Thus, building PTs’ self-efficacy to deliver telehealth care and build effective patient-therapist relationships, particularly with phone-only communication, was a key determinant. We trained PTs in MAPS which combines motivational interviewing and cognitive behavioral techniques to help patients set and achieve personally meaningful goals [51]. Motivational interviewing is a person-centered communication strategy well-suited to phone delivery [69–71] and circumstances where the patient and PT have different cultural backgrounds [72, 73], possibly due to motivational interviewing’s collaborative, nonjudgmental nature which may reduce risks for implicit biases by providers [74].

There are important limitations to note in our IM process. We did not pilot our implementation strategies or resources before beginning our hybrid clinical trial. As a hybrid trial, a secondary goal of the BeatPain study is the evaluate implementation. We intended to conduct needs assessments and patient interviews in-person, but COVID restrictions necessitated using zoom instead.

## Conclusion

BeatPain Utah is a hybrid type I trial evaluating effectiveness and implementation outcomes [36]. IM provided a systematic, theory-driven process to develop and evaluate implementation strategies. Assessment of implementation outcomes will allow us to evaluate the success of our implementation strategies for future trials and clinical applications.

## Data Availability

Not applicable.

## Abbreviations

CFIR: Consolidated Framework for Implementation
CHC: Community health center
EBI: Evidence-based intervention
EHR: Electronic health record
IM: Implementation mapping
LBP: Low back pain
MAPS: Motivation and problem-solving
PT: Physical therapist
SCT: Social cognitive theory
